# A modified technique for mechanical isolation of stromal vascular fraction yields increased final product volume and high viable nucleated cells count

**DOI:** 10.1002/jeo2.70378

**Published:** 2025-07-27

**Authors:** Trifon Totlis, Panagiotis‐Konstantinos Emfietzis, Argiro Niti, Vlasiοs Achlatis, Lucienne A. Vonk, Ioannis Terzidis, Kokkona Kouzi‐Koliakou

**Affiliations:** ^1^ Thessaloniki Minimally Invasive (The‐MIS) Orthopaedic Center, St. Luke's Hospital Thessaloniki Greece; ^2^ School of Medicine, Faculty of Health Sciences Aristotle University of Thessaloniki Thessaloniki Greece; ^3^ Biohellenika Biotechnology Company Thessaloniki Greece; ^4^ Xintela AB Lund Sweden; ^5^ Department of Orthopaedics University Medical Center Utrecht, Utrecht University Utrecht the Netherlands

**Keywords:** gene expression, osteoarthritis, platelet rich plasma, stem cells, stromal vascular fraction

## Abstract

**Purpose:**

Purpose of the present study is to report a modified protocol for stromal vascular fraction (SVF) isolation from abdominal fat, analyse its cellular composition and gene expression profile, and verify the safety and feasibility of the harvesting‐preparation technique and subsequent intra‐articular knee injection.

**Methods:**

The SVF was obtained after mechanical dissociation of the autologous harvested adipose tissue. It was combined with autologous platelet‐rich plasma and subsequently intra‐articularly injected into both knees of patients with osteoarthritis. Part of the SVF solution was used to analyse its cellular composition via flow cytometry and its gene expression profile via real‐time polymerase chain reaction. Any postinjection complications were documented.

**Results:**

Twenty‐three patients (10 female; 62.4 ± 8.4 years old) were enrolled. Posttreatment adverse events were mild and spontaneously resolved. Patients needed 3.7 ± 1.3 days for their knee to feel the same as before the injection. No patient provided less than 60 mL of lipoaspirate. Per 1 mL of SVF the total amount of viable nucleated cells was 49.7 ± 22.9 × 10^6^ on average, including 44.5 ± 23.2 × 10^6^ CD90+/CD105+ adipose‐derived stem cells, 0.54 ± 0.18 × 10^6^ hematopoietic stem cells, 2.71 ± 1.18 × 10^6^ pericytes and 1.88 ± 0.64 × 10^6^ endothelial cells. The polymerase chain reaction analysis revealed the following average values: Transforming growth factor beta 7.94 ± 3.31; vascular endothelial growth factor 12.43 ± 5.20; interleukin‐10 7.54 ± 2.88; octamer‐binding transcription factor 3/4 2.65 ± 1.69; interleukin‐1 beta 5.45 ± 3.27 and ki‐67 6.01 ± 3.65.

**Conclusion:**

A modification of an existing mechanical SVF preparation technique was introduced. The technique was feasible, safe and yielded a substantial volume of SVF (2.5–5 mL). The SVF obtained had a high cellular composition. Age, gender and body mass index (BMI) did not affect the cell count, but elder patients presented a decreased composition in cytokines and growth factors.

**Level of Evidence:**

Level V.

**Abbreviations**
ACP: autologous conditioned plasma
ADSCs: adipose derived stem cells
AD‐SVF: adipose‐derived stromal vascular fraction
AE: adverse event
BMAC: bone marrow aspirate concentrate
BMI: body mass index
BMSCs: bone marrow derived stem cells
CD: cluster of differentiation
GAPDH: glyceraldehyde‐3‐phospate dehydrogenase
HSCs: hematopoietic stem cells
ICF: informed consent form
IL‐10: interleukin‐10
IL‐1β: interleukin‐1 beta
IRB: Institutional Review Board
MSCs: mesenchymal stem cells
NSAIDs: nonsteroid anti‐inflammatory drugs
OA: osteoarthritis
Oct3/4: octamer‐binding transcription factor 3/4
PI: principal investigator
PRP: platelet‐rich plasma
qRT‐PCR: quantitative reverse transcription polymerase chain reaction
rt‐PCR: real‐time polymerase chain reaction
SAE: serious adverse event
SD: standard deviation
SVF: stromal vascular fraction
TGF‐β: transforming growth factor beta
tSVF: tissue‐SVF
VAS: visual analog scale
VEGF: vascular endothelial growth factor

## INTRODUCTION

Cell treatments have evolved to be a promising technique for the management of knee osteoarthritis (OA), especially with intra‐articular injection which is a relatively easy procedure that could also be used in ambulatory care. The injected cells include mesenchymal stem cells (MSCs) which have high plasticity, self‐renewal capabilities and immune‐suppressive and anti‐inflammatory properties [14]. Notably, the application of cell therapies has consistently been shown to be safe [43], while they do not preclude additional future therapy in case of treatment failure.

The origin of cell treatments can vary, but the two most common types of cells used for knee OA are bone marrow derived stem cells (BMSCs) (or bone marrow aspirate concentrate, BMAC) and adipose‐derived stem cells (ADSCs) or adipose‐derived stromal vascular fraction (AD‐SVF) [35]. An advantage of the BMAC and the AD‐SVF is that they can be used for autologous point‐of‐care treatment compared to the culture‐expanded MSCs, which is a two‐stage treatment [13, 30]. The SVF is a heterogeneous product that contains ADSCs, macrophages, blood cells, pericytes, fibroblasts, endothelial cells and their progenitors. Some of the acknowledged SVF actions can be attributed to the viable ADSCs found in the SVF, while others could be associated with the paracrine effect of all the cells and growth factors that are present in SVF [3].

Several point‐of‐care adipose‐derived cell therapies for knee OA are available, which are easily delivered due to their autologous nature and minimal manipulation required for the preparation. These treatments seem to be effective in pain reduction and functional improvement [1, 13], but little is known about their effect on cartilage regeneration and disease modification in clinical practice. A major limitation is the high heterogeneity in the manufacturing technique and subsequently in the composition of each product. Composition analysis of each treatment may be useful to better understand their mechanism of action.

Labarre and Zimmermann [26] recently utilised the technique for the preparation of SVF injection, which is available from Arthrex and was originally described by Stevens et al. [45]. Furthermore, they used a cell counter and reported an average of 44.9 million nucleated cells injected, without analysing the cell population with flow cytometry.

The purpose of the present study is to report a modified protocol for SVF isolation from patients' abdominal adipose tissue and analyse the cellular composition and the expression of genes in the final product. Furthermore, the study aims to verify the safety and feasibility of the reported SVF harvesting and preparation technique and subsequent intra‐articular injection of the SVF in combination with platelet rich plasma (PRP), for usage in the treatment of knee OA.

## MATERIALS AND METHODS

### Study design and participants

This is a single‐centre study carried out in St. Luke's Hospital, Thessaloniki, Greece. The AD‐SVF solution that was ultimately mixed with platelet‐rich plasma (PRP) before injection, was obtained after mechanical dissociation of the adipose tissue of patients with knee OA. A part of the SVF solution was not injected into the patient's knee. Instead, it was kept in optimal conditions and was used later to analyse its cellular composition via flow cytometry and, also, to construct a gene expression profile of the solution via real‐time polymerase chain reaction (rt‐PCR).

Out of all the patients that the principal investigator (PI) examined, those with knee OA were informed of our available studies and were asked for their participation. If the PI‐suggested and patient‐desired treatment was the combined injection of adipose‐derived SVF and PRP, then these patients were informed in detail of the present study. If they agreed to participate, a thorough check of the inclusion and exclusion criteria took place. The study obtained an Institutional Review Board (IRB) approval from the scientific committee of St. Luke's Hospital, Thessaloniki on 6th August 2021. All the patients that participated in the study, have signed an Informed consent form (ICF). The study was performed in accordance with the principles of the Helsinki Declaration and was performed in a way that always prioritised the patients’ rights, safety and well‐being.

The inclusion criteria for this study were men or women, aged 18–80 years old, with the ability to comprehend instructions provided by the medical staff, with a diagnosis of grade II, III or IV, bilateral knee OA based on Kellgren–Lawrence classification, confirmed by an X‐ray provided by the patient in the initial assessment. The patients' initial knee pain must be self‐evaluated by the patient at four or greater on a 10‐point visual analog scale (VAS). The patient must have adequate body fat content so that liposuction is feasible. Some of the exclusion criteria were a history or current autoimmune disorders/immunosuppressive status or malignancy, current pregnancy or current breastfeeding for women of childbearing potential, grade IV OA of joints other than the knee, current abdominal hernia, recent severe trauma of the knee, axial deviation of the suffering knees >15° (varus or valgus knees), history of cancer affecting the musculoskeletal system, signs of local or systemic infection, history of allergy to any substances used within the treatment, treatment with intra‐articular injections in the knee <3 months before the injection of this study, history of surgery on the suffering knee <1 year before the initial assessment, use of anticoagulants/antiplatelet/antithrombotic drugs <7 days before the injection, use of nonsteroid anti‐inflammatory drugs (NSAIDs) < 7 days before the injection and use of systemic corticosteroids <14 days before the injection.

### SVF production technique

**Figure 1:**
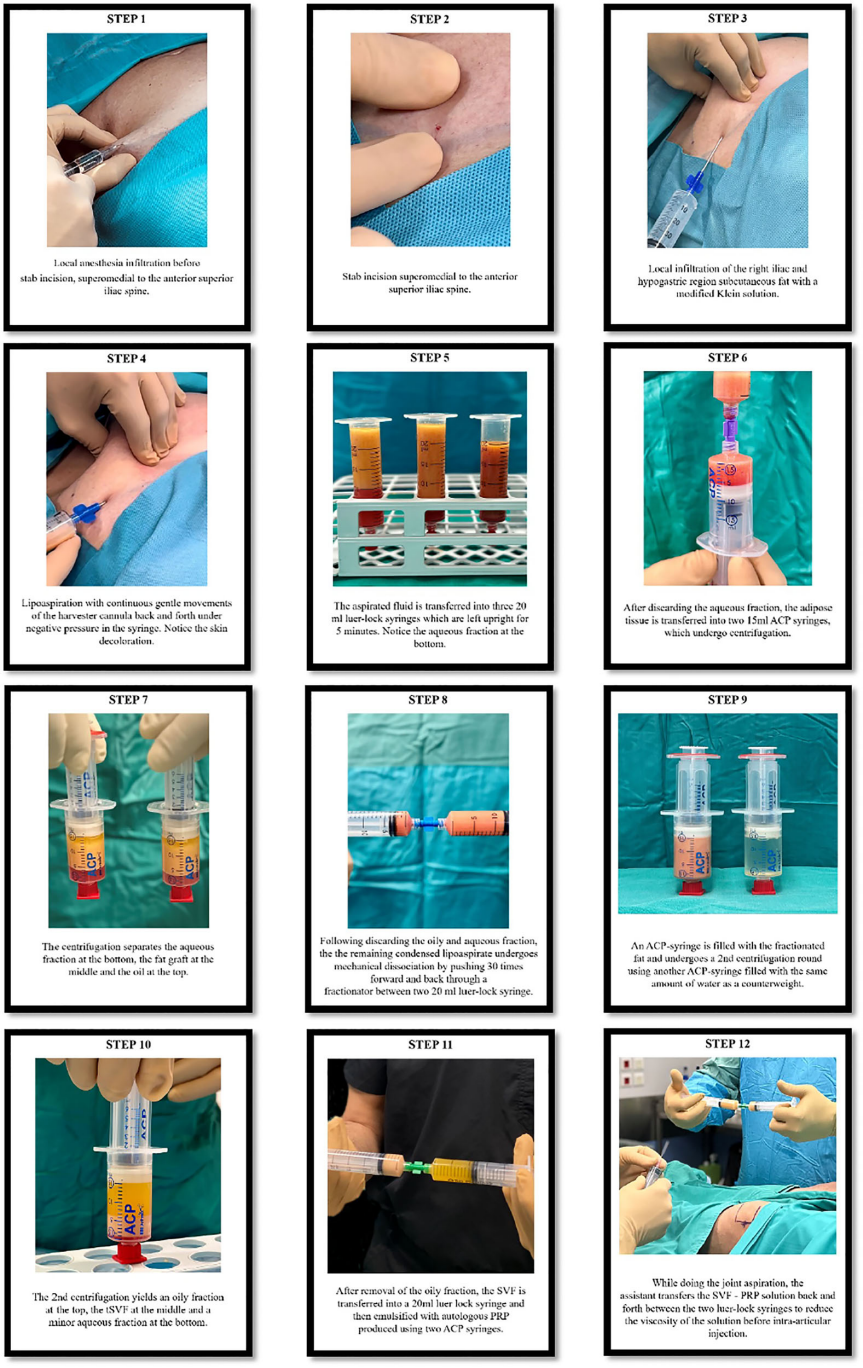
Step‐by‐step stromal vascular fraction preparation technique.

The current technique is a modified version of the technique reported by Stevens et al. [45] and was designed to optimise the final volume and cell count of tissue‐SVF (tSVF) derived from adipose tissue. A step‐wise overview of the procedure is presented in Figure 1.

The patient was placed in 30° beach‐chair position which is more comfortable for the patient and the abdominal adipose tissue was gathered at the hypogastric region. Under local anaesthesia, a stab incision was done, superomedial to the anterior superior iliac spine and a mosquito was used to distend the subcutaneous tissue. Local infiltration of a certain triangular region including the ipsilateral iliac region and hypogastric region was performed using the ACA‐Kit ABS‐10056 infiltration canula (Arthrex GmbH). For the infiltration approximately 180 mL (range 160–200 mL, depending on the quantity of the patient's abdominal adipose tissue) of a modified Klein solution (500 mL NaCl 0.9%, 50 mL lidocaine 2%, 1 mL adrenaline 1 mg/mL and 1 mL bicarbonate 4%) was used. Twelve minutes following the infiltration, the corresponding skin region was decolourised, and the skin temperature at palpation was reduced as a result of the adrenaline‐related vasoconstriction. Subsequently, 60–70 mL of lipoaspirate was harvested from the same region using a carraway harvester canula and a 30 mL Vaclock syringe from the ACA‐Kit ABS‐10056 (Arthrex GmbH). When one side was not enough to obtain 60–70 mL of lipoaspirate, the same procedure was performed on the contralateral side of the abdomen to obtain the desired quantity of lipoaspirate. The aspirated fluid was transferred into three 20 mL luer‐lock syringes which were left upright for 5 min. As a result, the aqueous fraction was gathered at the bottom of each syringe and subsequently discarded. Then, 30 mL of the adipose tissue that was left into the three luer‐lock syringes was transferred into two separate ACP (Arthrex GmbH) syringes (15 mL of adipose tissue in each syringe) using a 1‐hole disposable fractionator (1 × 2.4 mm hole, luer‐to‐luer transfer, ACA‐Kit ABS‐10056, Arthrex GmbH). For the fractionation of adipose tissue procedure, both syringes were placed into the sterilised buckets of a swing‐out rotor centrifuge (Rotofix 32A, Hettich GmbH). Following the first round of centrifugation for 4 min at 2500 rpm (769 g), the oily and aqueous fractions were discarded from both ACP syringes and the remaining condensed lipoaspirate was transferred from both ACP syringes into one 20 mL luer‐lock syringe. Subsequently, this syringe was connected to another empty 20 mL luer‐lock syringe using a 1‐hole disposable fractionator in between (1 × 1.4 mm hole, luer‐to‐luer transfer, ACA‐Kit ABS‐10056, Arthrex GmbH). The mechanical dissociation was conducted by pushing the condensed lipoaspirate 30 times forward and back through the fractionator in a nonmanipulative way. Then, 15 mL of the lipoaspirate was transferred back into an ACP‐syringe, put into the sterilised capsule and centrifuged for a second time for 4 min at 2500 rpm (769 g) using another ACP‐syringe filled with water as a counterweight. This yielded a final oily fraction at the top that could be easily removed with the small inner syringe leaving a fraction of 2.5–5 mL tSVF in the large outer ACP syringe. Beneath the tSVF fraction, an aqueous fraction with a minor pellet fraction was located. A small portion (0.5–1 mL) of this tSVF solution was transferred into a 1 mL luer‐lock syringe and sent to the laboratory for analysis. The remaining 2–4 mL tSVF was transferred into a 20 mL luer‐lock syringe. 8−12 mL ACP‐PRP was obtained from 30 mL of patient's venous blood using two ACP double syringe systems (Arthrex GmbH ACP System) according to the manufacturers' protocol. The 8–12 mL of PRP were transferred in another 20 mL luer‐lock syringe. The two luer‐lock syringes were connected with a 1.2 mm fractionator (1 × 1.2 mm hole, luer‐to‐luer transfer, ACA‐Kit ABS‐10056, Arthrex GmbH) and the tSVF was emulsified with the PRP. The emulsified solution was transferred forward and back between the two luer‐lock syringes in a nonmanipulative way, several times until the moment of injection to reduce the viscosity of the solution and avoid any obstruction within the needle during the intra‐articular injection. Half of the final solution was intra‐articularly injected to each knee of the patient using a 19G needle. Thereby, 1–2 mL of SVF and 4–6 mL of PRP were injected in each knee joint (5–8 mL total volume per knee).

### Flow cytometry

The 1 mL luer‐lock syringe containing 0.5−1 mL tSVF fraction was sent to the Laboratory (BioHellenika), where 0.5 mL tSVF was used for cell count, cell viability and subsequently the cluster of differentiation (CD) surface marker expression analysis via flow cytometry. The SVF solution was centrifuged at 2900 rpm for 20 min to obtain a high‐density SVF pellet, followed by enzymatic lysis of red blood cells. The staining for flow cytometry analysis was performed with the following monoclonal antibodies: CD90 (FITC)/CD105 (PE) for ADSCs, CD45 (FITC)/CD34 (PE) for hematopoietic stem cells (HSCs), and CD146 (PE)/CD34 (FITC) (EXBIO) for pericytes and endothelial cells enumeration, respectively. 7‐AAD (Beckmann Coulter Inc.) was used for dead and live cell discrimination in order to assess the cellular viability. The analysis results were obtained on a FACSCalibur device (Becton Dickinson, BD) and analysed with CellQuest Pro6 software.

### Gene expression analysis

The gene expression profile of the tSVF was examined in 16 samples to evaluate the potential involvement of the SVF in various biological pathways including anti‐inflammatory, pro‐inflammatory, angiogenic, cellular proliferation and totipotency pathways [29, 32, 33, 34, 42, 46]. RNA was extracted using the Nucleospin RNeasy Mini Kit (MACHEREY‐NAGEL), according to the manufacturer's instructions. RNA concentration and purity analyzed using a NanoDropND‐1000 UV‐Vis Spectrophotometer. Gene expression levels of Transforming Growth Factor beta (TGF‐β), interleukin 10 (IL‐10), vascular endothelial growth factor (VEGF), ki‐67 and octamer‐binding transcription factor 3/4 (Oct3/4) and IL‐1 beta (IL‐1β) were determined using the KAPA SYBR FAST qPCR Kit (Kapa Biosystems), in a Rotor‐Gene 6000 operating system (Corbett machine, KARYO). The expression data of target genes were normalised relative to the expression levels of the housekeeping gene glyceraldehyde‐3‐phospate dehydrogenase (GAPDH).

### Safety evaluation

The safety of the reported SVF harvesting and preparation technique and subsequent SVF plus PRP injection was evaluated with documentation of any intraoperative and postoperative complications within the first 2 weeks posttreatment. During those 2 weeks, patients were encouraged to communicate with the study team and report anything that felt unusual so that we can report it in our findings as an adverse event (AE)/serious adverse event (SAE). Before the injection, all patients had been informed that the intra‐articular injection of SVF and PRP is followed by mild sensitivity of the knee joint. This symptom was not documented as an AE. The day that it was resolved was used as an evaluation tool. Namely, the patients were asked to notice the day that their knee feeling was the same as before the injection. At 2 weeks after the injection, all patients were contacted and asked to report any complications and how many days after the injection their knee feeling had returned to the feeling before the treatment. Furthermore, five random samples were tested in the Laboratory for aerobic and anaerobic contaminants using the Bactec method (Bactec 9120, Becton Dickinson).

### Statistical analysis

Descriptive statistics were performed to determine the mean values and standard deviation (SD). Furthermore, analyses were performed to examine any correlations between cell counts and gene expression with age, body mass index (BMI) and sex of the patients. If the two variables tested for significant correlation were both normally distributed, then the Pearson correlation test was used. If any of the two variables or both of them were not normally distributed, then the Spearman correlation test was applied. For the correlations between cell counts and sex, the statistical test used was the Independent Samples *t*‐test (if the continuous variable was normally distributed) or the Mann–Whitney *U* test (if the continuous variable was not normally distributed). For the statistical analysis, the SPSS software (v 27.0) was used. The level of significance was set at *p* < 0.05.

## RESULTS

### Study participants

A total number of 23 patients (10 female and 13 male) with a mean age of 62.4 (SD: 8.4) years and a mean BMI of 30.8 (SD: 2.9)—mean height of 169.9 (SD: 8.3) cm and mean weight of 88.9 (SD: 9.6) kg—were enrolled in this study. Flow cytometry was performed in tSVF samples from all 23 patients and PCR analysis in 16 of them. All patients completed the study.

### Safety

During the study, 34 AEs were reported by the patients. Three of them were a minor headache that was treated with paracetamol. These AEs were not considered related to the SVF harvesting technique or the injection. Twenty‐six of the AEs reported by 13 patients were redness (haematoma) and sensitivity in the harvesting area (iliac and hypogastric region). Five AEs reported by five patients were only sensitivity in the harvesting area. These 31 harvesting procedures related to AEs were considered mild, and they were treated with ice. All of them were resolved within 10 days after the injection. None of these AEs were deemed serious and none of them was related to the intra‐articular injection of the treatment solution. All five samples tested for sterility were found sterile.

Patients reported that 3.7 (SD: 1.3, range 1–6) days were needed for their knee to feel the same as before the injection. The detailed values reported by patients can be found in Table 1.

**Table 1:** Detailed demographics and cell populations count of the study cohort.

Patient ID	Sex	Age	BMI	CD90 + CD105 + ADSCs (×10^6^/mL)	HSCs (×10^6^/mL)	Pericytes (×10^6^/mL)	Endothelial cells (×10^6^/mL)	Total viable nucleated cells (×10^6^/mL)	Safety (days)
001	F	65	35.6	76.4	0.5	1.78	1.96	80.64	2
002	M	82	40.1	27.6	0.9	3.02	2	33.52	3
003	F	54	33.2	36.8	0.56	1.82	2.56	41.74	4
004	M	58	30.0	42	0.31	2.28	1.6	46.188	3
005	M	48	28.9	56	0.64	1.74	2.68	61.06	5
006	M	68	29.4	24	0.84	0.18	0.17	25.19	3
007	M	68	30.4	82	0.42	4.24	3.44	90.1	3
008	F	47	34.4	104	0.36	1.88	2.04	108.28	6
009	F	61	28.7	58	0.5	1.8	1.84	62.14	5
010	F	68	32.0	34	0.22	3.74	2	39.96	4
011	M	50	33.0	39.4	0.74	4.94	1.88	46.96	5
012	M	63	28.3	6.4	0.54	3.54	1.2	11.68	4
013	F	61	32.0	42	0.28	4.9	1.9	49.08	3
014	M	62	29.7	9.4	0.62	1.62	2.1	13.74	3
015	M	69	29.3	30	0.3	1.9	2.3	34.5	2
016	M	71	30.1	17	0.42	3.48	1.04	21.94	1
017	F	55	31.6	51	0.54	1.88	1.22	54.64	4
018	F	64	29.7	57	0.63	2.95	2.14	62.72	5
019	M	58	26.2	41.4	0.37	3.12	1.95	46.84	3
020	F	77	30.5	37.5	0.65	3.08	2.36	43.59	6
021	M	67	28.7	29.8	0.58	4.05	1.35	35.78	3
022	M	57	27.0	58.9	0.74	2.14	1.54	63.32	4
023	F	63	30.1	63.4	0.7	2.2	1.95	68.25	4
Mean value		62.4	30.1	44.5	0.54	2.71	1.88	49.7	3.7
SD		8.6	3.1	23.2	0.18	1.18	0.64	22.9	1.3

Abbreviations: ADSCs, adipose derived stem cells; BMI, body mass index; F, female; HSCs, hematopoietic stem cells; M, male; SD, standard deviation.

### Feasibility

In 21/23 cases (91.3%), one‐sided liposuction was enough to obtain 60–70 mL of lipoaspirate. In 2/23 cases (8.7%), the same liposuction procedure was performed on the contralateral side of the abdomen to obtain the desired quantity of lipoaspirate. No patient provided less than 60 mL of lipoaspirate using the current lipoaspiration technique at the iliac and hypogastric region.

### Cellular composition

The average percentage of viable cells was 91.53% with a range of 82.74%–95.98%. The total amount of SVF‐derived viable nucleated cells was 49.7 × 10^6^ (SD: 22.9 × 10^6^) per mL. The CD90+/CD105+ ADSCs count had a mean value of 44.5 × 10^6^ (SD: 23.2 × 10^6^) per mL of SVF solution. The HSCs count had a mean value of 0.54 × 10^6^ (SD: 0.18 × 10^6^) per mL of SVF solution. The pericytes cell count had a mean value of 2.71 × 10^6^ (SD: 1.18 × 10^6^) per mL of SVF solution. The endothelial cells count had a mean value of 1.88 × 10^6^ (SD: 0.64 × 10^6^) per mL of SVF solution. The different cell counts for each patient can be found in Table 1.

### PCR analysis

The PCR analysis revealed the gene expression profile of the SVF solution that was harvested from the patients. The values are the relative expression levels normalised to the expression level of GAPDH: TGF‐β mean value 7.94 (SD: 3.31); VEGF mean value 12.43 (SD: 5.20); IL‐10 mean value 7.54 (SD: 2.88); Oct3/4 mean value 2.65 (SD: 1.69); Il‐1β mean value 5.45 (SD: 3.27) and ki‐67 mean value 6.01 (SD: 3.65). The values of the gene expressions in the SVF solution of each patient can be found in Table 2.

**Table 2:** Detailed values of the quantified normalised gene expression levels in the SVF solution of each patient.

Patient ID	TGF‐β	VEGF	IL‐10	Oct3/4	IL‐1β	ki‐67
001	13.42	18.44	10.52	3.45	8.12	11.07
002	2.83	4.29	7.45	5.78	4.44	0.17
003	8.83	16.54	12.44	2.15	7.96	5.45
004	10.22	18.77	6.45	2.745	9.24	6.84
005	12.57	13.72	7.54	1.45	8.54	10.07
006	3.41	8.54	2.1	0.78	2.22	2.96
007	4.57	4.77	3.41	2.45	7.47	3.06
008	10.5	16.74	8.85	3.44	9.22	10.2
009	4.45	15.74	7.45	1.45	8.54	10.85
010	7.58	3.48	4.36	5.96	2.45	5.31
011	12.67	19.11	10.11	0.77	8.02	7.15
012	5.44	12.47	8.74	2.96	1.11	6.67
013	7.96	11.14	5.49	1.55	0.24	9.25
014	7.52	8.79	5.78	2.04	3.71	2.04
015	6.78	12.74	11.45	0.66	4.9	0.27
016	8.21	13.67	8.45	4.82	1.06	4.73
Mean value	7.94	12.43	7.54	2.65	5.45	6.01
SD	3.31	5.20	2.88	1.69	3.27	3.65

Abbreviations: IL‐10, interleukin 10; IL‐1β, interleukin 1 beta; Oct3/4, octamer‐binding transcription factor ¾; SD, standard deviation; TGF‐β, transforming growth factor beta; VEGF, vascular endothelial growth factor.

### Correlations

**Figure 2:**
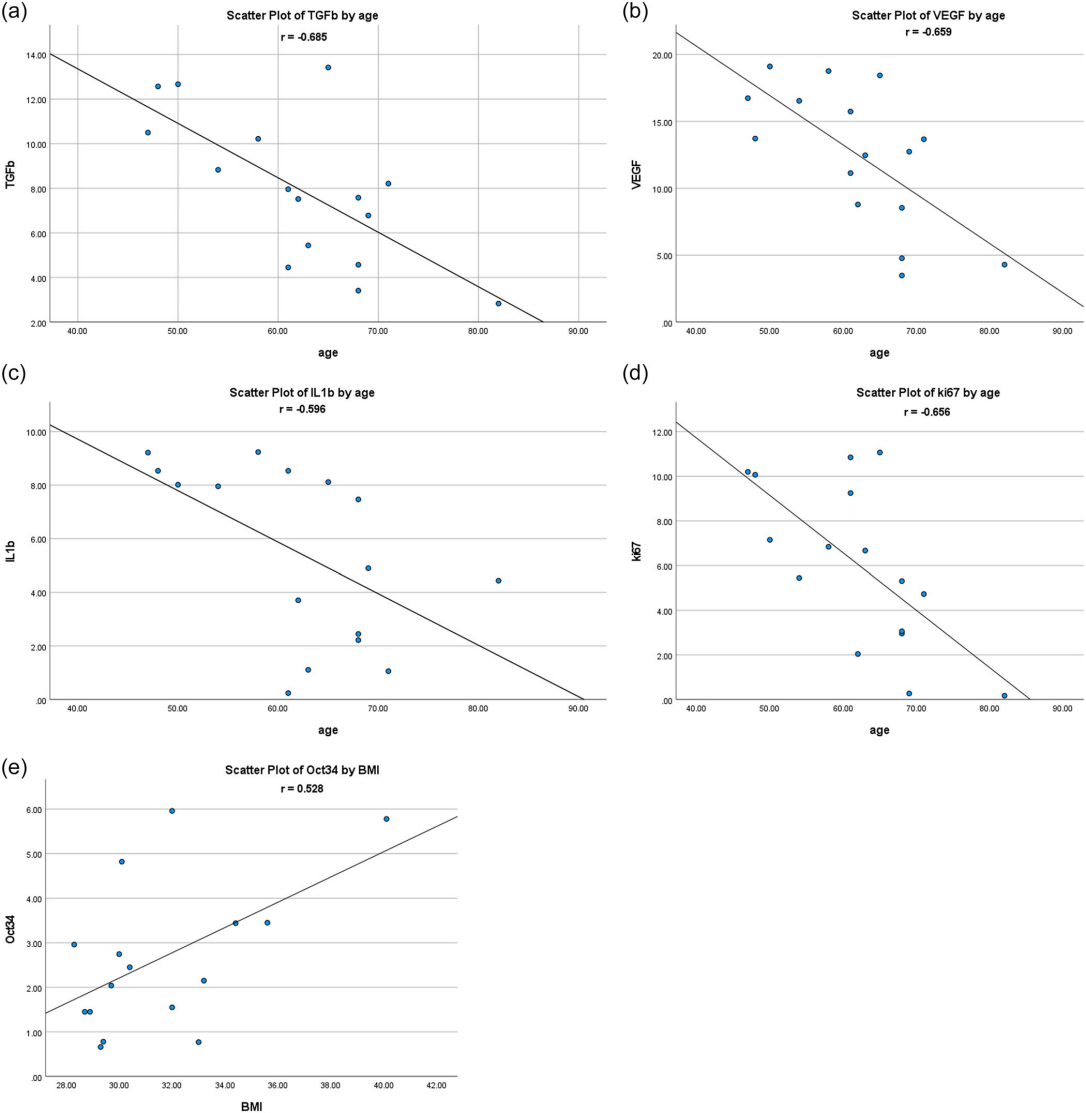
Scatter plots of transforming growth factor beta (TGF‐β, a), vascular endothelial growth factor (VEGF, b), interleukin 1 beta (IL‐1b, c), ki67 (d) by age and octamer‐binding transcription factor 3/4 (Oct34, e) by body mass index with the best fit line as reference and Pearson's correlation coefficient.

**Figure 3:**
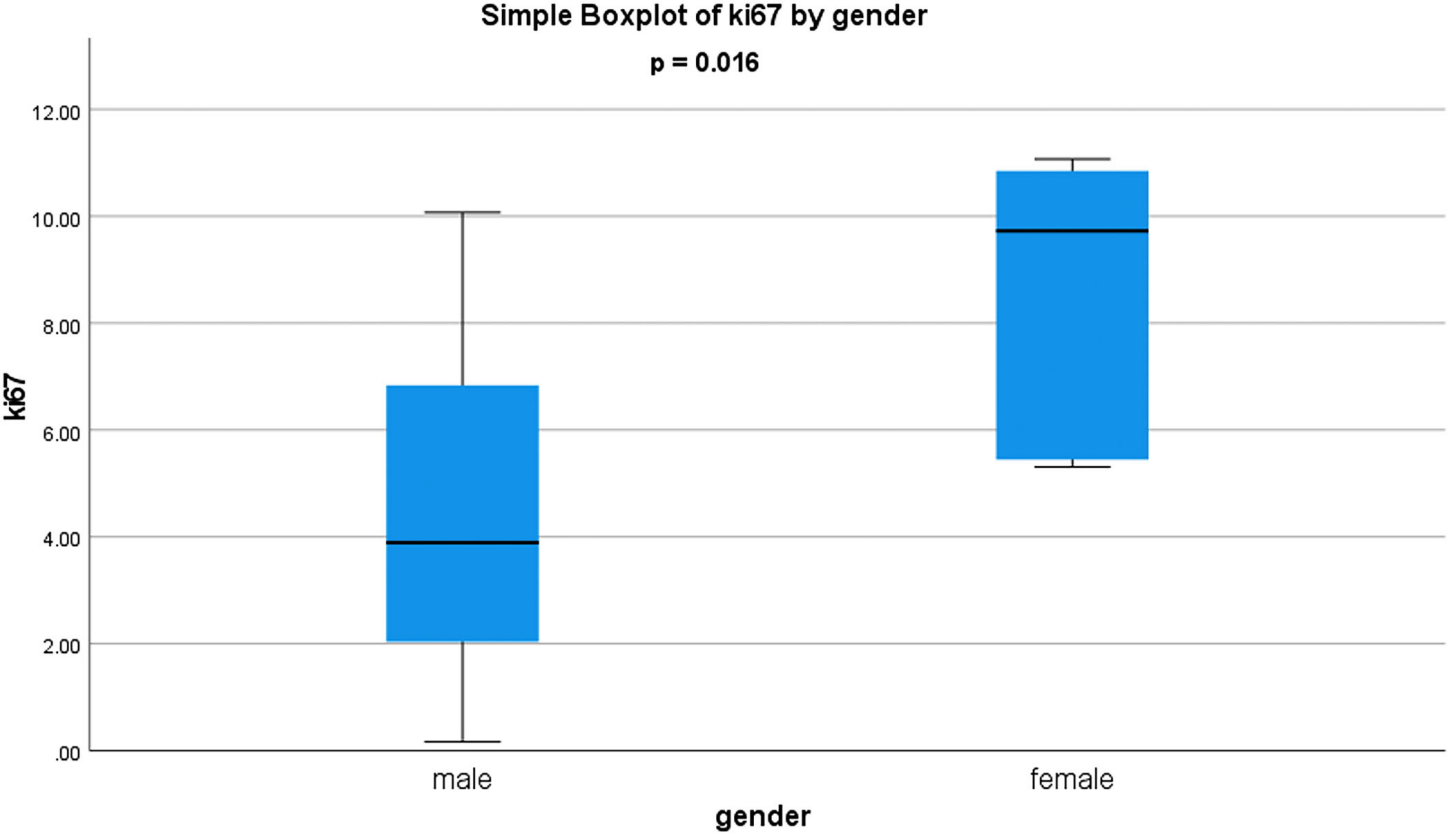
Side‐by‐side boxplot of ki‐67 by sex (*p* = 0.016).

No statistically significant correlations were found between the different cell populations of the SVF solution and the patients' age, BMI and sex. The detailed results of the correlations tests can be found in Table 3. On the other hand, the age of the patients was significantly correlated with the quantitative gene expression of TGF‐β (*r* = −0.685, *p* = 0.003, Figure 2a), VEGF (*r* = −0.659, *p* = 0.006, Figures 2b), IL‐1β (*r* = −0.596, *p* = 0.015, Figure 2c) and ki‐67 (*r* = −0.656, *p* = 0.006, Figure 2d) in the SVF solution. Specifically, older age was correlated with decreased transcriptional expression levels of TGF‐β, VEGF, IL‐1β and ki‐67. BMI was found to be significantly correlated with the quantitative gene expression of Oct3/4 (*r* = 0.528, *p* = 0.035, Figure 2e) in the patients' SVF. Higher BMI was correlated with an increased transcriptional expression level of Oct3/4. The sex of the patients was significantly correlated with the quantitative gene expression of ki‐67 (*p* = 0.016, Figure 3) in the SVF solution. Namely, female sex was correlated with an increased transcriptional expression level of ki‐67. The detailed results of the correlations tests can be found in Table 3.

**Table 3:** *p* values of the statistical correlation tests (Pearson or Spearman) among the variables of the study cohort.

	Age	BMI	Sex
Flow cytometry			
CD90+CD105+ADSCs	0.065 (*r* = 0.390)	0.607 (*r* = 0.113)	0.243
HSCs	0.484 (*r* = 0.154)	0.816 (*r* = −0.051)	0.332
Pericytes	0.518 (*r* = 0.142)	0.703 (*r* = 0.084)	0.717
Endothelial cells	0.723 (*r* = −0.078)	0.182 (*r* = 0.288)	0.454
PCR analysis			
TGF‐β	0.003** (*r* = **−0.685)**	0.761 (*r* = 0.083)	0.443
VEGF	0.006** (*r* = **−0.659)**	0.735 (*r* = −0.092)	0.477
IL‐10	0.278 (*r* = −0.289)	0.376 (*r* = 0.237)	0.504
Oct3/4	0.062 (*r* = 0.476)	0.035* (*r* = **0.528)**	0.542
IL‐1β	0.015* (*r* = **−0.596)**	0.676 (*r* = 0.113)	0.566
ki‐67	0.006** (*r* = **−0.656)**	0.804 (*r* = −0.067)	0.016*

*Note*: The number inside the parentheses is the Pearson's or Spearman's correlation coefficient (*r*). *p < 0.05, **p < 0.01. Values in bold represent statistically significant findings.

Abbreviations: ADSCs, adipose derived stem cells; BMI, body mass index; HSCs, hematopoietic stem cells; IL‐10, interleukin 10; IL‐1β, interleukin 1 beta; Oct3/4, octamer‐binding transcription factor ¾; TGF‐β, transforming growth factor beta; VEGF, vascular endothelial growth factor.

## DISCUSSION

The most important finding of the present study was that the current modified technique for tSVF production yields a substantial volume of SVF (2.5–5 mL) composed by a high amount of viable nucleated cells (49.7 × 10^6^/mL), including 44.5 × 10^6^/mL CD90+/CD105+ ADSCs, 2.71 × 10^6^/mL pericytes, 1.88 × 10^6^/mL endothelial cells and only a low proportion of HSCs (0.54 × 10^6^). Furthermore, any concerns with regard to the feasibility and safety of the modified technique, because of the greater volume of lipoaspirate and tSVF intra‐articular injection, were eliminated.

There are two primary categories of SVF distinguished by their processing methods. Cellular SVF is isolated through enzymatic lysis of adipose tissue, which is considered as a form of substantial tissue manipulation. In contrast, tSVF is solely produced by mechanical fragmentation, including filtration and/or centrifugation, which are considered minimal manipulation [10]. In the present study, the lipoaspirate underwent only centrifugation and mechanical fragmentation through transfer devices before the injection to the patient. Moreover, human tissues are divided into four categories: connective, muscle, neural and epithelial tissue. The adipose, tendons, ligaments, cartilage and joints are tissues belonging to the connective tissue. Therefore, some authorities consider intra‐articular injection of adipose‐derived tSVF homologous use, while others require injection into the infrapatellar fat pad to comply with homologous use.

Many studies have investigated the composition of SVF. The nucleated cells found in SVF may include ADSCs, endothelial cells, pericytes, HSCs, fibroblasts, macrophages and lymphocytes among others [6, 7]. ADSCs are a heterogeneous population of stromal cells isolated from adipose tissue with regenerative potential, and characteristics similar to that of MSCs [6]. Particularly, they are characterised by their ability to differentiate into adipogenic, osteogenic and chondrogenic lineages (trilineage differentiation) [23, 31, 37].

There is great variability among published studies regarding the surface markers used and the terminology of cells found in the SVF. Cohen et al. and Tiryaki et al. defined the ADSCs as CD45−CD90+/CD73+CD90+ cells, whereas Van Dongen et al. defined them as CD45–CD90+CD105+ cells [9, 47, 50]. Jang et al. used the CD34+CD31−CD146− markers to identify the adipose‐derived stromal cells and the CD45−CD31−CD90+CD105+ markers to identify the MSCs in adipose‐tissue SVF samples [23]. On the other hand, Copcu and Oztan used the CD14‐FITC, CD90‐FITC, CD146‐PE, CD34‐ECD, CD45‐PC5 markers to isolate a cell population from SVF and called them “TOST (total stromal) cells” [11]. However, the International Federation for Adipose Therapeutics and Science (IFATS) and the International Society for Cellular Therapy (ISCT) have established minimal criteria to define the MSCs derived from adipose tissue [7]. According to Bourin et al. MSCs are characterised by the following markers CD90+CD105+CD73+CD45−CD34−CD14 or CD11b−CD79a or CD19−HLADR− [7].

The current study used flow cytometry and certain markers to identify four different cell subtypes in the tSVF. The total number of these cells was 49.7 × 10^6^ (SD: 22.9 × 10^6^) per mL of SVF solution on average and they may be called SVF‐derived viable nucleated cells. Only a small proportion of these cells was HSCs (0.54 × 10^6^/mL), pericytes (2.71 × 10^6^/mL) and endothelial cells (1.88 × 10^6^/mL). An amount of 44.5 × 10^6^ cells per ml were CD90 and CD105 positive. The CD90+CD105+ cells' ability for trilineage differentiation has been approved in the literature [22, 23, 31, 54]. Therefore, these cells may be called CD90+CD105+ ADSCs to determine that this is the CD90 and CD105 positive subpopulation of the ADSCs which exist into the SVF. A more extensive characterisation of those cells according to Bourin et al. would be necessary to identify the number of MSCs per ml within the SVF samples [7]. According to the literature, SVF has a fraction of ADSCs, but since they express different cell markers and possess different properties, they are not strictly defined as MSCs [36]. They are widely recognised as MSC‐like cells [28, 39, 44]. SVF contains a diminutive percentage of MSCs, estimated at 2%–10% of the SVF [18]. Therefore, only a small proportion of the 49.7 × 10^6^ total viable cells identified in the current study are likely MSCs.

Labarre and Zimmerman [26] and Jang et al. [23] reported 44.9 × 10^6^ and 42.9 × 10^6^ nucleated cells in tSVF, respectively. In both studies, this number of cells represent the total amount of nucleated cells measured with a cell counter or a cellometer, instead of flow cytometry. Labarre and Zimmerman [26] found a wide range of cell count (6.61 × 10^6^ − 98.5 × 10^6^) and Jang et al. [23] observed a high SD of the measured cells (31.6 × 10^6^). These findings are in accordance with the high SD found in the present study underscoring the interdonor variability in the cells yield and SVF composition [49]. Other studies on tSVF found lower values of cell count into the final product [9, 11, 47, 48, 51]. However, this difference probably reflects the more extensive characterisation used by other authors compared to the current study. An extensive review of clinical studies that used autologous and allogenic ADSCs obtained from the infrapatellar fat pad, gluteus and abdomen reported a range from 10^6^ cells per mL in SVF to 10^8^ cells per mL in cultured ADSCs [36].

This study involved several modifications to the original protocol as outlined by the manufacturer [5]. Stevens et al. originally described the method for isolating this SVF product [4, 45]. According to these descriptions, the tumescent fluid for local infiltration was 30 mL of lidocaine 2% mixed with epinephrine 1:200.000 plus 3 mL of sodium bicarbonate, diluted into 500 mL NaCl 0.9%. In the current study, the composition of the fluid for local infiltration was 500 mL NaCl 0.9%, 50 mL lidocaine 2%, 1 mL adrenaline 1 mg/mL and 1 mL bicarbonate 4%. In the original technique, 30 mL of lipoaspirate was harvested 15 min after the infiltration, whereas in the present study 60−70 mL of lipoaspirate was collected 12 min after the infiltration and an extra step was added. The 60−70 mL aspirated fluid was transferred into three 20 mL luer‐lock syringes which were left upright for 5 min. As a result, the aqueous fraction was gathered at the bottom of each syringe and subsequently discarded. Then, 30 mL of the adipose tissue was transferred into two ACP syringes (15 mL each syringe). The rest of the procedure was in accordance with the manufactures protocol. With this modification, the 30 mL lipoaspirate which undergoes the first centrifugation contains clearer adipose tissue with less aqueous fraction. Consequently, after the 1st centrifugation a total volume of more than 15 mL of dry fat graft is obtained which subsequently undergoes fragmentation through a 1.4 mm luer‐to‐luer transfer fractionator and then a 2nd centrifugation. At the end, the SVF volume with the current protocol is 2.5–5 mL compared to 1–1.5 mL originally reported [5, 45]. This modification was designed to increase the final SVF volume and cells administrated into the joint. With the current technique, obtaining 2.5–5 mL of SVF allows for intra‐articular injection of at least 1–2 mL of SVF in each knee joint, which means 49.7–99.4 × 10^6^ viable nucleated cells on average. According to the literature, the volume of lipoaspirate may positively influence the number of cells obtained in the SVF [25].

SVF exerts its therapeutic effects primarily through the secretion of paracrine factors, which are signalling the host cells, thus stimulating and augmenting endogenous regenerative processes [53]. Growth factors, particularly VEGF, are key factors released by stromal cells that significantly enhance angiogenesis [27]. Stem and progenitor cells within SVF exhibit immunomodulatory, anti‐inflammatory and anti‐apoptotic properties. This is achieved by stimulating the expression of IL‐10 and TGF‐β [8, 53] and suppressing the expression of IL‐1β [15]. Using qRT‐PCR analysis, Choi et al. [8] reported that enzymatic harvesting of SVF resulted in higher expression of VEGF‐A (2.2‐fold) and IL‐10 (83.2‐fold) compared to the housekeeping gene GAPDH. The present study findings are consistent with this observation, but a higher increase for VEGF‐A (12.43‐fold) and a lower increase for IL‐10 (7.54‐fold) were observed. Similarly, Jin et al. [24] employed enzymatic harvesting and demonstrated that human SVF expressed VEGF‐A at a level higher than GAPDH (approximately 30‐fold) using qRT‐PCR.

The current study found a mean ki‐67 expression value of 6.01, indicating cellular proliferation within the isolated SVF, which may be beneficial for stimulating the endogenous regenerative processes. These values do not constitute overexpression of ki‐67, thus eliminating any concerns for induction of uncontrolled cellular proliferation. Oct3/4 is reported as a well‐known marker of totipotency, due to its vital role in maintenance of self‐renewal and undifferentiated state in stem cells [32]. To assess the risk of tumorigenicity of SVF, Van Pham et al. [52] investigated the Oct3/4 expression in human SVF compared to embryonic stem cells. They found a much lower expression of this gene in the SVF samples compared to embryonic stem cells, which is in line with the very low mean value of 2.65 that was found in the present study, thereby supporting the safety profile of the SVF.

This study found no significant correlations between patients' age, BMI and sex with the various cell counts within the isolated SVF solution. Labarre and Zimmerman [26] reported no significant correlation with sex, and Faustini et al. [19] observed no significant influence of BMI or age on the SVF cellular composition. On the other hand, other authors found a significant positive correlation between BMI [2] and younger age [16] with the number of cells within the SVF. Dykstra et al. [17] reported a potential inverse association between MSCs count and BMI and no correlation between age and MSCs or endothelial progenitor cell counts. The reason behind this discrepancy among studies is not known but could be related to various technical steps. In conclusion, the literature is inconsistent with regard to the association between patient demographics and cell count in the SVF.

The literature is scarce about patients' characteristics which may affect the growth factor and cytokine production or the expression of their corresponding genes in the SVF. The relevant studies are few, heterogenous in terms of the adipose tissue source and inconsistent regarding their findings [12, 20, 21]. The present study found that older age was significantly correlated with decreased expression of TGF‐β, VEGF, IL‐1β and ki‐67. Furthermore, higher BMI was significantly associated only with increased Oct3/4 expression, and female sex only with increased ki‐67 expression. IL‐10 expression was not correlated with age, BMI, or sex. Clinical translation of those findings is not easy. Patients' age was the only characteristic associated with many genes (four out of the six examined). Consequently, it can be assumed that SVF from older patients might have a decreased composition in cytokines and growth factors. The lower expression of TGF‐β and VEGF might indicate that SVF from older patients have less regenerative properties, while lower ki‐67 indicates less proliferative capacity and lower IL‐1β suggests a lower pro‐inflammatory effect. However, further research is needed to validate this observation.

The lipoaspiration was feasible in all patients of the study cohort without facing any difficulties. However, skinny patients with not enough body fat to perform liposuction were not considered candidates for this treatment. This evaluation was subjective and conducted by the principal investigator. According to the relevant literature, this issue is a limitation of the adipose‐derived cell treatments [53]. Thereby, if the literature guidelines are taken into consideration in patient selection, then the current modified technique for tSVF production which needs 60–70 mL abdominal fat aspiration is feasible. When one side is not enough to obtain this amount of lipoaspirate, then the same procedure may be performed on the contralateral side of the abdomen. The duration of the whole procedure was between 40 and 60 min, depending on the need for contralateral lipoaspiration and unilateral or bilateral knee injection.

Any posttreatment adverse events were mild and spontaneously resolved within less than 10 days. All patients felt an increased sensitivity on their knees after injection, which was expected from previous cases (before initiation of the study). Thereby, instead of reporting the proportion of patients having this symptom, the duration of this symptom was evaluated. It was resolved between 1 and 6 days and according to this finding, all patients were prescribed physical therapy to begin at 7 days postinjection. Nguyen et al. reported four cases with AEs which were hypertension, dyspnoea, chest pain and urinary retention. These AEs were not associated with SVF liposuction and injection as it was the case with the three patients who reported a headache in the present study [38]. Russo et al. described two cases of organised haematoma following abdominal fat harvesting, attributed to coagulation issues and a slim physique and recurrent effusions in one case [40]. Proper patient selection and avoiding too superficial lipoaspiration could be helpful to prevent large haematomas in the abdominal region. Santoprete et al. observed knee swelling and pain in 7% of patients during the first seven days of postprocedure [41]. No study documented the days those patients needed to feel their knees the same as before the injection. All procedures were performed in the operating room taking into consideration all proper sterilisation and disinfection techniques, both during the lipoaspirate and the intra‐articular knee injection. The tSVF solution was tested in the laboratory for sterility and found sterile.

The present study has certain limitations. There was a high variability in the results obtained both in the cell count and the PCR analysis, which is reflected by the high standard deviation. The interdonor variability in the cells yield and SVF composition is well known in the literature [49]. Age, BMI and sex did not significantly affect the number of cells, but a larger sample might be preferrable to assess the role of these factors. A weak point of this study is the limited characterisation of the SVF derived cells subtypes. A more extensive characterisation would be necessary to define the MSCs count into the final product. Actually, only the CD90 and CD105 positive subpopulation of the ADSCs which exist into the SVF was currently determined. However, it is noteworthy that the CD90+CD105+ cells' ability for trilineage differentiation has been approved in the literature [22, 23, 31, 54]. Furthermore, the CD45 and CD34 positive cells subpopulation was also identified in the current study SVF samples and the corresponding proportion was low. Another limitation of the present study is the absence of adipocytes identification in the final SVF product. Uguten et al. reviewed several tSVF isolation procedures and underscored the ability of each system to reduce the lipoaspirate volume isolating tSVF as pure from adipocytes as possible [49]. They concluded that the superior mechanical isolation method, in terms of SVF purity, might be centrifugation, shuffling adipose tissue forward and backward through small holes and again centrifugation [49]. This particular sequence was followed in the current study. Last limitation is the absence of postinjection clinical outcomes. At the moment, the authors considered important to investigate the safety and feasibility of the treatment with the modified SVF preparation technique and document the efficacy of the current modification in terms of SVF volume, cell count and gene expression. Increasing the number of cases would be useful to obtain reliable correlations between cytometry and PCR findings with postinjection clinical outcomes in the future.

## CONCLUSION

A modification of an existing mechanical SVF preparation technique was introduced in the present study. The technique was feasible, safe and yielded a substantial volume of SVF (2.5–5 mL). The SVF obtained had a high cellular composition, including CD90+CD105+ ADSCs, pericytes and endothelial cells. Age, gender and BMI did not affect the cell count, but SVF from elder patients presented a decreased composition in cytokines and growth factors.

## AUTHOR CONTRIBUTIONS

**Trifon Totlis**: Conceptualisation; methodology; data curation; visualisation; writing—review and editing; supervision. **Panagiotis‐Konstantinos Emfietzis**: Data curation; writing—original draft. **Argiro Niti**: Data curation; visualisation; writing—original draft. **Vlasiοs Achlatis**: Data curation; formal analysis. **Lucienne A. Vonk**: Visualisation; writing—review and editing; supervision. **Ioannis Terzidis**: Writing—review and editing; supervision. **Kokkona Kouzi‐Koliakou**: Conceptualisation; methodology; supervision.

## CONFLICT OF INTEREST STATEMENT

The authors declare no conflicts of interest.

## ETHICS STATEMENT

The study was performed in accordance with the principles of the Helsinki Declaration and was performed in a way that always prioritised the patients' rights, safety and well‐being. The study obtained an Institutional Review Board (IRB) approval from the scientific committee of St. Luke's Hospital, Thessaloniki on August 6, 2021. All the patients that participated in the study, have signed an Informed Consent Form (ICF).

## Data Availability

The raw data supporting the findings of this study are provided in the manuscript tables.
